# Plans of Hospitals

**Published:** 1909-03-20

**Authors:** 


*To the Editor of* The Hospital.

### PLANS OF HOSPITALS.

*To the Editor of* The Hospital.

Sir,—In your recent issues there has been published a series of plans, in which the points of the compass are referred to as "unfortunately omitted."

The aspect of a hospital building is such an important matter that it should, I think, be made a rule that no plans will be published unless the north point is indicated thereon. I feel sure that if this view were adopted the authors of the plans would take care that the necessary information should be supplied. Yours faithfully,

R. L. C.

23 Throgmorton Street, E.C., March 8.

